# Overcoming *tet*(X)-harboring tigecycline resistance: a study on the efficacy of tigecycline-apramycin combinations

**DOI:** 10.3389/fmicb.2024.1502558

**Published:** 2024-12-23

**Authors:** Juan Liu, Si-Lin Zheng, Jing-Jing Wu, Mei Zheng, Da-Tong Cai, Yan Zhang, Jian Sun, Xiao-Ping Liao, Yang Yu

**Affiliations:** ^1^Guangdong Laboratory for Lingnan Modern Agriculture, National Risk Assessment Laboratory for Antimicrobial Resistance of Animal Original Bacteria, College of Veterinary Medicine, South China Agricultural University, Guangzhou, China; ^2^State Key Laboratory for Animal Disease Control and Prevention, South China Agricultural University, Guangzhou, China; ^3^Guangdong Wenshi Dahuanong Biotechnology Co. Ltd., Yunfu, Guangdong, China; ^4^Animal Laboratory Center, Guangzhou University of Chinese Medicine, Guangzhou, China; ^5^Guangdong Provincial Key Laboratory of Veterinary Pharmaceutics Development and Safety Evaluation, South China Agricultural University, Guangzhou, China

**Keywords:** *tet*(X)-harboring *Acinetobacter* spp., tigecycline, apramycin, synergistic combination therapy, mouse thigh infection model

## Abstract

**Introduction:**

The emergence of the wide variety of novel tigecycline resistance *tet*(X) variants, including *tet*(X3), *tet*(X4), *tet*(X5), and *tet*(X6), has raised a serious threat to global public health and posed a significant challenge to the clinical treatment of multidrug-resistant bacterial infections.

**Methods:**

In this study, we evaluated the synergism of tigecycline combining with other antibiotics as a means of overcoming the *tet*(X)-mediated resistance in *Acinetobacter* spp. Antibiotic synergistic efficacy was evaluated through *in vitro* chequerboard experiments, time-kill assays and dose–response curves. The *in vivo* synergistic effect of the combination was confirmed in a mouse model of thigh with neutrophilic granulocyte reduction. Additionally, combinations were tested for their ability to prevent high-level tigecycline-resistant mutants.

**Results:**

We found that the combinations of tigecycline with apramycin exhibited synergistic activity against *tet*(X)-harboring Acinetobacter spp. with FICI of 0.088. The MICTGC decreased more than 5 times in the presence of subinhibitory levels of apramycin. The combination showed in vitro synergism in time-kill assays and in vivo therapeutic effectiveness in the mouse thigh infection model.

**Discussion:**

This study shed light on the synergism of tigecycline in combination with apramycin which offers a viable therapeutic alternative for infections caused by *tet*(X)-harboring *Acinetobacter* spp.

## 1 Introduction

The rapid emergence and global dissemination of *Acinetobacter* spp. as a major nosocomial pathogen is remarkable, particularly in intensive care units where it can cause illnesses such as pneumonia, tracheobronchitis, meningitis, endocarditis, peritonitis, skin and soft tissue infection and urinary tract and bloodstream infections (Glew et al., 1977; Malta et al., 2020). In recent decades, infections caused by *Acinetobacter* spp. have increased in frequency and severity due to the abuse of antibiotics and antimicrobial resistance (Zweier, 2014). Currently, the options for combatting multidrug-resistant *Acinetobacter* spp. using antibiotics are limited.

Universally, treatment regimens for *Acinetobacter* spp. infections rely on colistin and tigecycline, either alone or in combination with carbapenems (Cheng et al., 2021). Tragically, these last line agents for treating MDR-bacterial infections are increasingly challenged by the emergence and spread of antimicrobial resistance genes such as *tet*(X4)/(X5), *mcr* and *bla*_NDM_/*bla*_KPC_ (Cui C. et al., 2020; Li et al., 2020). Tigecycline, approved for clinical use in 2005, can overcome most of the resistance mechanisms related to tetracyclines (Koomanachai et al., 2009; Rubinstein and Vaughan, 2005; Sun et al., 2019). It provides an opportunity for its use in nosocomial infections where resistance is more likely to occur, especially as the incidence of multidrug-resistant (MDR) *Acinetobacter* spp. is rising (Lee et al., 2017). However, this last-resort antibiotic may become ineffective due to new tigecycline resistance *tet*(X) variants that include *tet*(X3), *tet*(X4), *tet*(X5), and *tet*(X6). Recent studies have highlighted the urgent need for new treatment options (Tang et al., 2021; Cui Z. H. et al., 2020). In such situations, tigecycline-based regimens are alternative treatments against MDR bacteria (Hornsey and Wareham, 2011; Tamma et al., 2012; Nulsopapon et al., 2021) and it is crucial to explore effective drug combination to reduce the required tigecycline dosage.

Combination therapy is a method that involves the use of two or more active antibiotics in conjunction. This approach minimizes the dosage of toxic drugs and reduces the frequency of drug resistance, achieving more significant biochemical effects than monotherapy (Fitzgerald et al., 2006). For instance, a combination regimen of tigecycline and amikacin efficiently inhibited development of tigecycline resistance and successfully suppressed emergence of resistant populations (Moland et al., 2008). Tigecycline activity was also increased both *in vitro* and *in vivo* when used in combination with zidovudine against *E. coli* that carried *tet*(X) and *mcr-1* (Zhou et al., 2020). Additionally, the use of apramycin in a mouse model of *A. baumanii* was effective when the AUC/MIC ratio was >50 and *C*_max_/MIC was 10 or higher (Meyer et al., 2014).

Here we explore the combination of tigecycline and apramycin. There are only a limited number of studies exploring antibiotic synergy and its mechanisms. Therefore, we sought to explore whether apramycin in combination with tigecycline can be effective against multidrug-resistant (MDR) *Acinetobacter* spp. strains harboring *tet*(X).

## 2 Methods and materials

### 2.1 Bacterial profiling and antimicrobial susceptibility

A total of nine *Acinetobacter* isolates carrying different *tet*(X) variants were separated from human, pig, migratory bird and environmental samples over the years and stored in a 30% glycerin broth at −80°C. The bacterial species were identified by matrix-assisted laser desorption/ionization time of flight mass spectrometry (MALDI-TOF, MS). The presence of *tet*(X) and co-harboring with *bla_NDM_* were identified by polymerase chain reaction (PCR) amplification and sequencing as previously reported (Cui C. et al., 2020). Antimicrobial susceptibility testing of tigecycline, meropenem, colistin, polymyxin B and the aminoglycosides amikacin, gentamicin, apramycin, kanamycin was performed by broth microdilution or disc diffusion methods according to EUCAST guidelines (EUCAST, 2023) And reference strain ATCC 25922 served as quality control.

### 2.2 Checkerboard assays and fractional inhibitory concentration index (FICI)

The checkerboard assay was used to test the joint antibacterial activity of tigecycline combined with seven antibiotics (see above). The reasons for these tests were as follows: (i) these drugs remained certain active against multidrug-resistant (MDR) *Acinetobacter* isolates (Fishbain and Peleg, 2010; Kang et al., 2017); (ii) some of them, like meropenem and colistin are still considered last resort treatments of infections caused by MDR Gram-negative bacteria (Hornsey and Wareham, 2011; Gordon and Wareham, 2010); (iii) few studies have evaluated the synergistic potential of tigecycline combinations against *Acinetobacter* spp. (Li et al., 2017).

In brief, isolate FS38-2 (MIC_TGC_ = 16 μg/mL and co-harboring *tet*(X3), *tet*(X6), and *bla*_NDM-1_ genes) was randomly selected for screening the synergic partners combining with tigecycline for the other seven antibiotics. The checkerboard assays of the potential drug combinations were performed against all nine isolates to further confirm the synergism. Bacterial cultures of 10^6^ CFU/mL were exposed to 2-fold dilutions of antibiotics with concentrations ranging from 1/8× to 8 × MIC in 96-well plates and incubated for 18 h at 37°C. The FICI was analyzed by the following equation:


$$\begin{array}{l} \mathrm{FICI}=\frac{\mathrm{MIC}\;\mathrm{of agent}\;A\;\mathrm{in combination}}{\mathrm{MIC}\;\mathrm{of agent}\;A\;\mathrm{alone}}\\ \;+\frac{\mathrm{MIC}\;\mathrm{of agent}\;B\;\mathrm{in combination}}{\mathrm{MIC}\;\mathrm{of agent}\;B\;\mathrm{alone}} \end{array}$$

where FICI ≤0.5 represents “synergism”; 0.5 < FICI ≤1 means “addition”; “indifferent” was defined when 1 < FICI ≤2; and FICI >2 denotes “antagonism” (Yu et al., 2018).

### 2.3 MICs_TGC_ in presence of apramycin

MIC values were determined using broth microdilution. Two-fold serial dilutions of tigecycline ranged from 0.25 to 128 μg/mL in 96-well microplates, respectively. The final concentration of apramycin was 0, 0.25, 0.5, 1, 2 μg/mL by adding 50 μL of different concentrations to each well. Furthermore, a purified single colony of isolated strains was picked up and emulsified to using MH broth medium. After that, 100 μL bacterial solution was added to make the final bacterial suspension contained an inoculum density of ~10^6^ CFU/mL. MICs_TGC_ in presence of apramycin were determined after incubation at 37°C for 18 h.

### 2.4 Time-killing assays

To further evaluate the synergistic effect of potential combinations, *tet*(X)-harboring *Acinetobacter* strains: *A. lwoffi* HZE30-1 and *A. indicus* WF106-1 (both harboring *tet*(X3), *tet*(X6), and *bla*_NDM-1_), *Acinetobacter* HNS1-2 carrying *tet*(X3) and *A. indicus* Q186-3 carrying *tet*(X4) and *tet*(X5) were randomly selected for *in vitro* time-killing assays. It was performed to evaluate the synergistic activity of sub-MICs of tigecycline and apramycin alone or in combination using LB broth medium with an initial inoculum of 10^6^ CFU/mL *Acinetobacter* strains as previously described. The prepared system was then incubated at 37°C and 180 rpm, and 100 μL of the suspension was diluted at 0, 3, 6, 9, and 24 h for bacterial counting.

### 2.5 Dose-response assays

As described previously (Yu et al., 2018), OD values at 8 h was selected to calculate the correlation between antimicrobial effects and dosage of drugs with formula using GraphPad Prism Version 8. The OD values for bacterial growth in the absence of antibiotics was used as the normalization standard. Results are shown as the mean value of all 9 isolates at a 95% CI. The dose–response correlation of tigecycline or apramycin was calculated with the equation:


$$E=E_{\text{max}}+\frac{E_{0}-E_{\text{max}}}{1+C^{\mathrm{HillSlope}}/I{C_{50}}^{\mathrm{HillSlope}}}$$

where *E* is the bacterial growth estimated as the OD_600_ normalized by that of control; *E_max_* is the maximal antimicrobial efficacy; *E_0_* is the bacterial growth in the absence of any antibiotics; *C* is the concentration of tigecycline or apramycin; *IC_50_* is the drug concentration demonstrating 50% of *E_max_*; Hillslope describes the curve gradient.

### 2.6 *In vivo* therapeutic experiment

**Figure 1:**
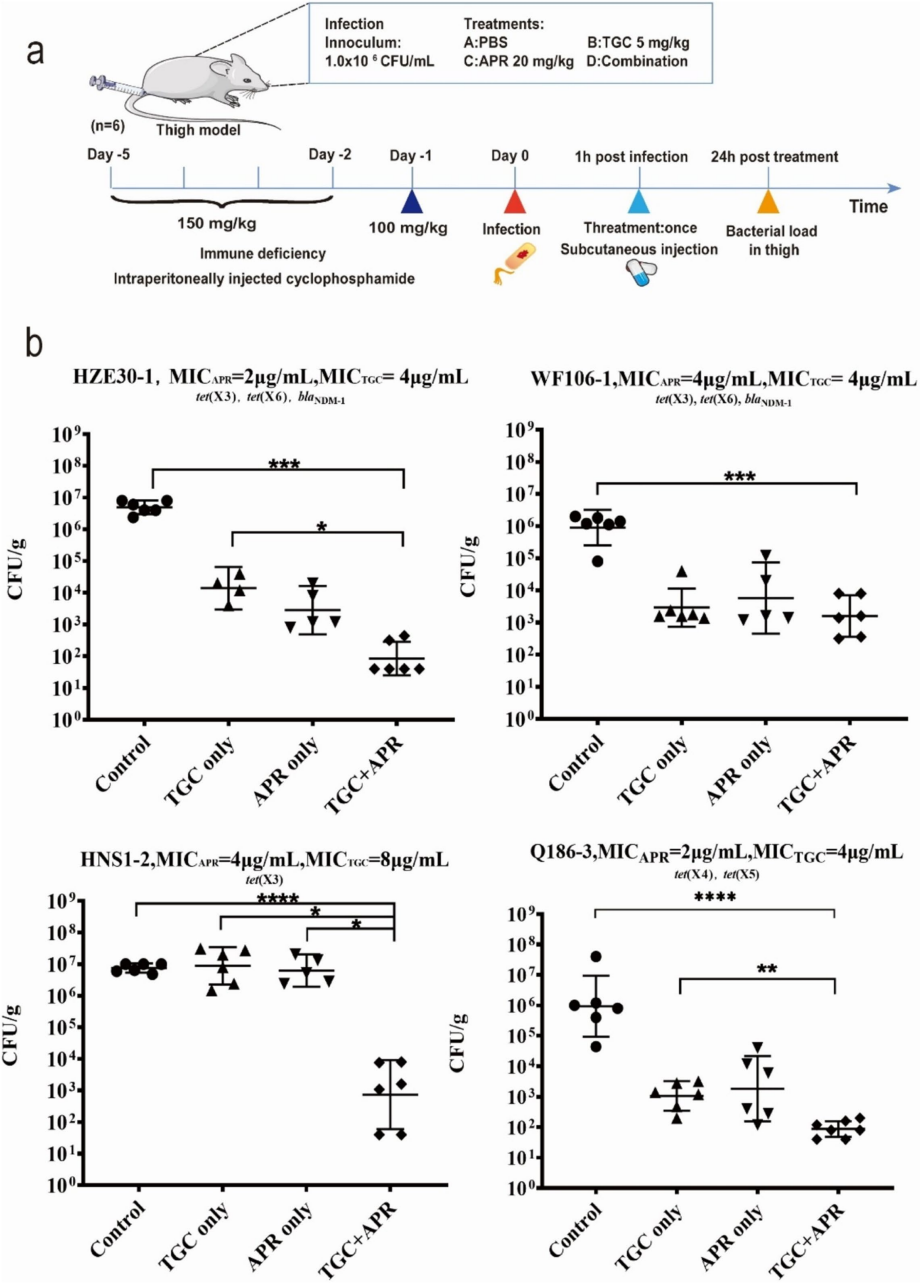
The *in vivo* synergy of tigecycline combining apramycin. **(A)** Scheme of the experimental protocol of animal trials. **(B)** Combination therapy of tigecycline plus apramycin in a mouse thigh model. *p < 0.05; **p < 0.01; ***p < 0.005; ****p < 0.001 by the one-way ANOVA.

As schematically illustrated in Figure 1A, the neutropenic mouse thigh model was employed for testing the *in vivo* synergistic efficacy of tigecycline plus apramycin. Specific-pathogen-free (SPF) female ICR mice weighing 25 ± 2 g at six-weeks-old (Hunan Silaikejingda Lab Animal, Hunan, China) were rendered temporarily neutropenic by immunosuppression with cyclophosphamide (Yuanye Biotechnology, Shanghai, China) at 150 mg/kg on the first four days and 100 mg/kg at the fifth day by intraperitoneal injection before bacterial inoculation (23). Four *tet*(X)-harboring strains used in the *in vitro* time-killing curve assays were employed here. The mid-log bacterial cultures were appropriately diluted with normal saline and the neutropenic mice (neutrophils ≤100/mm^3^) were then intramuscularly inoculated with 100 μL of bacterial suspension (10^6^ CFU/mL) into each posterior thigh muscle. After 1 h, infected mice were administered in the following manner: phosphate buffer saline (PBS) as control (Group I), tigecycline or apramycin alone as single-drug groups (Group II and III); and tigecycline combining with apramycin (Group IV). Tigecycline was administrated at 5 mg/kg subcutaneously, apramycin as 20 mg/kg subcutaneously and the injection volume was 100 μL for all drugs. The tigecycline doses were selected to mimic the pharmacokinetic profiles of recommended human clinical doses (Koomanachai et al., 2009; Meagher et al., 2005). In the case of apramycin, the doses were selected based on previous reports of pharmacodynamic effects on different MIC and preliminary experimental results (Meyer et al., 2014; Kang et al., 2018). The group IV was simultaneously injected subcutaneously with tigecycline 5 mg/kg/d and apramycin 20 mg/kg/d. Following 24 h of treatment, mice were sacrificed and thigh homogenates were sampled for bacterial burden quantification. Differences between groups were estimated by one-way analysis of variance (ANOVA) with 95% CI and graphs were generated using GraphPad Prism Version 8.

### 2.7 Prevention of high-level tigecycline-resistant mutants

To further investigate whether antibiotic combinations prevent the occurrence of high-level tigecycline-resistant mutants, nine strains were challenged with prolonged and repeated exposure to tigecycline alone and tigecycline plus apramycin. Approximately 10^6^ CFU/mL of *tet*(X)-carrying *Acinetobacter* isolates were challenged by tigecycline only and tigecycline plus 0.5 μg/mL apramycin. After incubating at 37°C for 18 h, cultures from each MIC well and the last well containing bacterial growth were mixed and challenged with the antibiotic groups described above. This protocol was repeated continuously for 14 days.

## 3 Results

### 3.1 Bacterial information

Table 1 showed the profiles of nine *tet*(X)-carrying *Acinetobacter* isolates, including *Acinetobacter haemolyticus*, *Acinetobacter* spp. and *Acinetobacter beijerinckii* isolates carrying *tet*(X3), *Acinetobacter lwoffii* and *Acinetobacter indicus* isolates co-harboring *tet*(X3), *tet*(X6) and *bla*_NDM-1_ and three *A. indicus* isolates harboring *tet*(X3), *tet*(X6)/*bla*_NDM-3_/*tet*(X4), and *tet*(X4)/*tet*(X5). All nine strains were resistant to tigecycline with MICs of 4–16 μg/mL but susceptible to colistin, polymyxin B, amikacin and apramycin. Four *bla*_NDM_-positive isolates exhibited resistance to meropenem and two *A. indicus* isolates were resistant to gentamicin.

**Table 1:** Molecular profiles and MICs of 9 *tet* (X)-harboring *Acinetobacter* strains.

Strain	Species	Resistance genes	MIC (μg/mL)							
			MEM	CS	PB	TGC	AMK	GEN	APR	KAN
JXZ5-1	*Acinetobacter haemolyticus*	*tet*(X3)	0.06	0.5	0.5	8	0.125	0.125	1	128
Z51-2	*Acinetobacter* spp.^*^	*tet*(X3)	0.06	1	1	16	0.125	0.125	2	2
HNS1-2	*Acinetobacter beijerinckii*	*tet*(X3)	0.015	1	1	8	0.5	128	4	256
HZE30-1	*Acinetobacter lwoffi*	*tet*(X3) *tet*(X6) *bla*_NDM-1_	16	0.5	1	4	8	0.125	2	64
MM119-1	*Acinetobacter indicus*	*tet*(X3) *tet*(X6) *bla*_NDM-3_	32	0.5	0.5	16	4	16	4	128
FS38-2	*Acinetobacter indicus*	*tet*(X3) *tet*(X6) *bla*_NDM-1_	4	1	1	16	4	32	2	256
WF106-1	*Acinetobacter indicus*	*tet*(X3) *tet*(X6) *bla*_NDM-1_	32	2	1	4	8	8	4	128
Q22-2	*Acinetobacter indicus*	*tet*(X4)	0.03	0.25	0.25	4	0.125	0.125	2	0.25
Q186-3	*Acinetobacter indicus*	*tet*(X4) *tet*(X5)	0.03	1	0.25	4	0.25	0.25	2	0.125

Resistance breakpoints refer to EUCAST guidelines (EUCAST, 2023). MEM, meropenem; CS, colistin; PB, polymyxin B; TGC, tigecycline; AMK, amikacin; GEN, gentamicin; APR, apramycin; KAN, kanamycin. ^*^Species of Z51-2 cannot be identified. Red indicates resistance to this drug.

### 3.2 *In vitro* checkerboard assays

**Figure 2:**
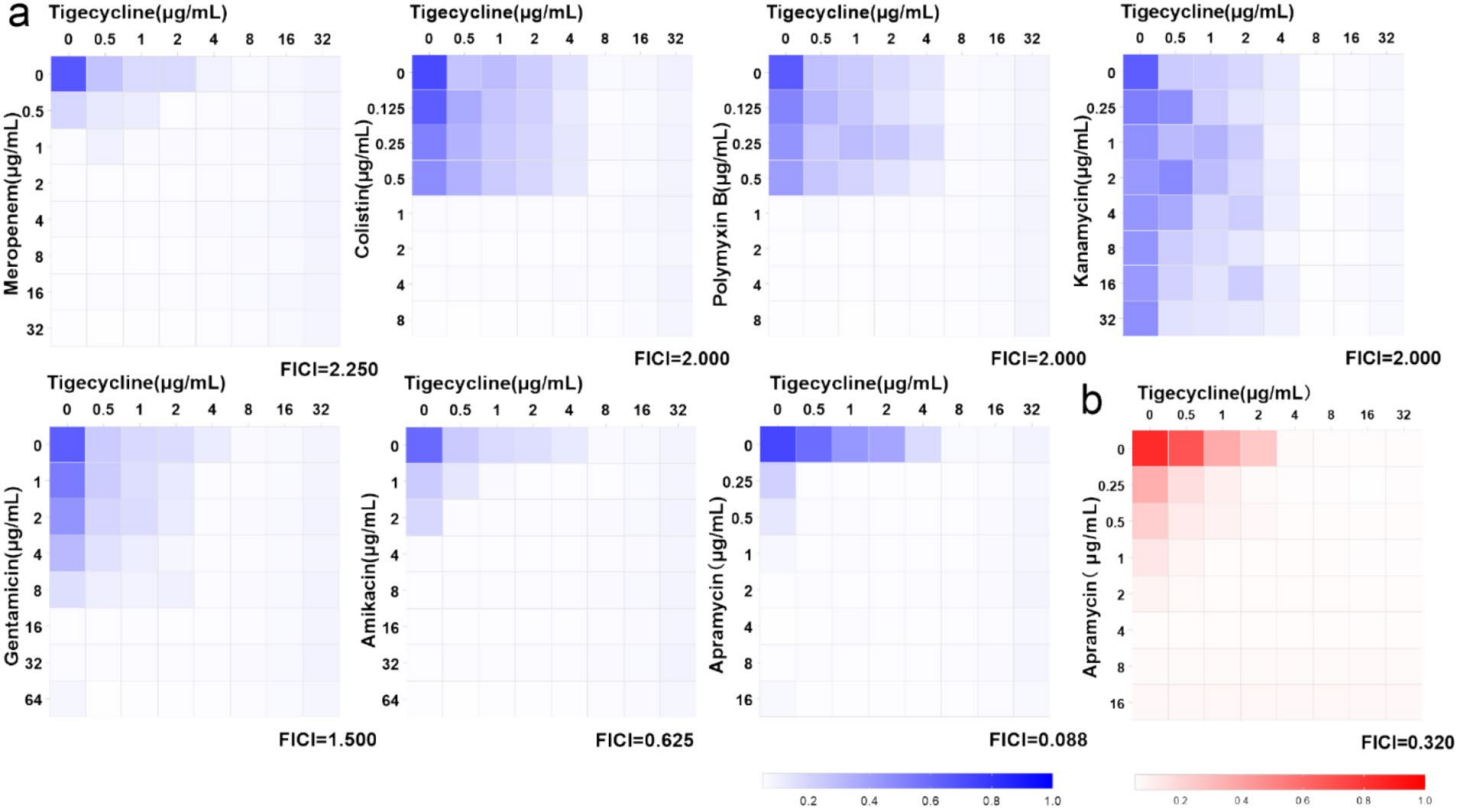
Heat-maps of checkerboard assays for tigecycline combined with the listed antibiotics. **(A)** Strain FS38-2 was used in the preliminary screen. **(B)** Synergistic effect of tigecycline plus apramycin against nine strains (FICI_average_ = 0.32, *n* = 9 biological replicates).

In Figure 2A, strain FS38-2 (*tet*(X3), *tet*(X6), and *bla*_NDM-1_) was selected for screening the synergism of tigecycline combined with other 7 antibiotics. The combination of tigecycline plus apramycin produced a significant antibacterial effect (FICI = 0.088). The other antibiotics with tigecycline showed ether indifferent or antagonistic interactions (FICI >1). Additionally, further evaluation of tigecycline plus apramycin was repeated against nine *Acinetobacter* strains and FICI_average_ of 0.32 was detected by checkerboard assays (Figure 2B; Supplementary Table S1).

### 3.3 MICs_TGC_ in presence of apramycin

**Figure 3:**
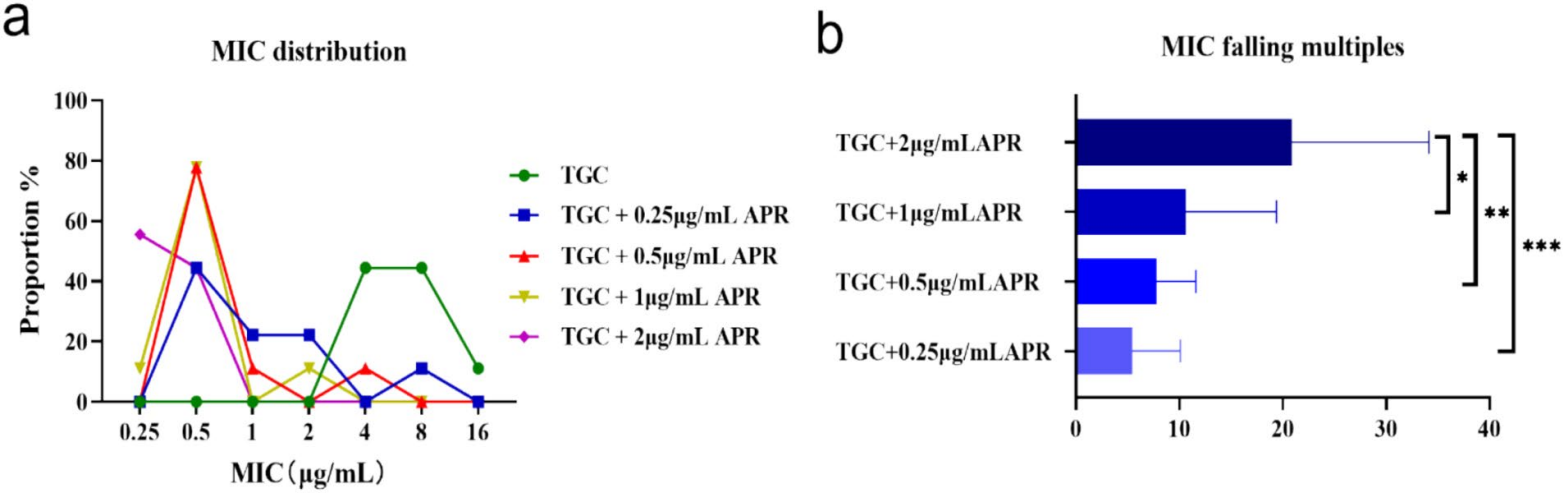
MIC changes of tigecycline against 9 *tet*(X)-harboring *Acinetobacter* strains when combined with apramycin at various concentrations. **(A)** MIC distributions of tigecycline alone or combined with different doses of apramycin. **(B)** MIC reductions of tigecycline in the presence of apramycin. *p < 0.05, **p < 0.01, and ***p < 0.001 by the one-way ANOVA. APR, apramycin; TGC, tigecycline; control, normal saline.

A combination of tigecycline and a series of sub-inhibitory concentrations of apramycin to determine whether the combination of the two is effective. As apramycin concentration increasing, the MICs_TGC_ distribution shifted towards lower MIC values of 0.25–0.50 μg/mL (Figure 3A). Over 5 times reduction of tigecycline MIC was observed in the presence of subinhibitory levels of apramycin. Notably, tigecycline MICs dropped more than 20 times in presence of 2 μg/mL apramycin on average (*n* = 9), which was significantly lower than 0.25 μg/mL apramycin (*p* < 0.001) (Figure 3B).

### 3.4 Time-kill assay

**Figure 4:**
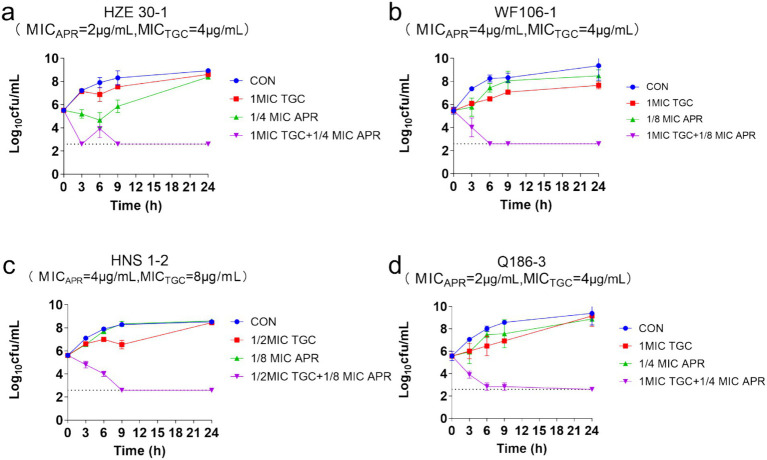
The time-killing curves of *Acinetobacter* strains treated with apramycin, tigecycline, and combined regimen.

The time-killing curves of *tet*(X)-harboring *Acinetobacter* strains treated with tigecycline plus apramycin were shown in Figure 4, and the grow of 4 *Acinetobacter* isolates (HZE 30-1, WF106-1, HNS1-2, Q186-3) were all inhibited with the delayed logarithmic phase and stationary phase. Synergistic effects for the combinations were determined in isolates HZE 30–1, WF106-1, HNS1-2, and Q186-3 with concentrations of 1MIC TGC + 1/4MIC APR, 1MIC TGC + 1/8MIC APR, 1/2MIC TGC + 1/8MIC APR, and 1MIC TGC + 1/4MIC APR, respectively. In Figure 4, none of the drugs alone could completely inhibit bacterial growth during the nine hours of exposure. On the contrary, the cell growth and proliferation were severely inhibited using the combined regimen after 9 h of incubation showing an effective synergism. After 24 h, more than 5 log_10_ reductions in colony count were detected in all combination groups.

### 3.5 Dose-response curves

**Figure 5:**
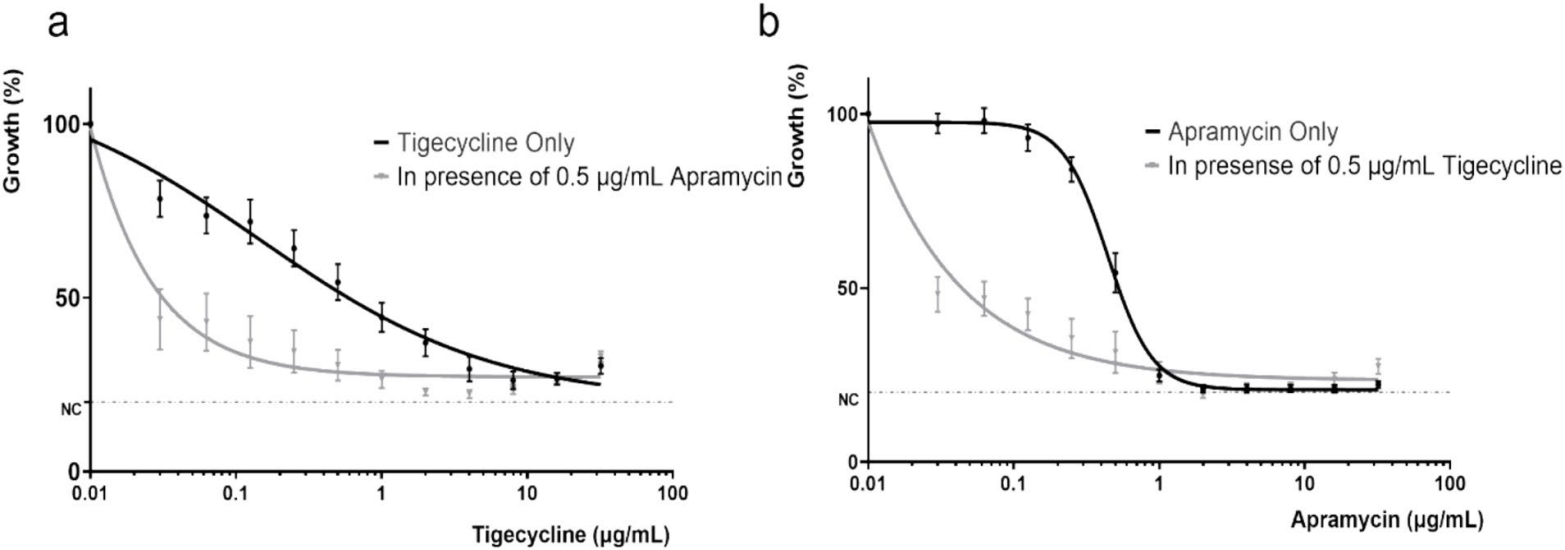
Dose response curves of different combinations of tigecycline and apramycin for *tet*(X)-harboring *Acinetobacter* strains. **(A)** Dose response curve of tigecycline alone and in combination with apramycin (0.5 μg/mL). **(B)** Dose response curve of apramycin alone and in combination with tigecycline (0.5 μg/mL). NC, negative control (minimum detection limit).

The concentration-effect relationship was fitted to a Hill-type equation in order to accurately determine the response to antibiotic challenge. Curves for tigecycline alone and tigecycline plus apramycin illustrated that greater inhibitory activity were obtained as the tigecycline concentration was increased (Figure 5A). Importantly, compared with the single-drug groups, the IC_50_ of two-drug groups (apramycin with 0.5 μg/mL tigecycline and tigecycline with 0.5 μg/mL apramycin) dropped to <0.0001 μg/mL (Table 2).

**Table 2:** Dose–response parameters of different combinations of tigecycline and apramycin against 9 *tet*(X)-harboring *Acinetobacter*.

Parameter	Tigecycline only	Tigecycline +0.5 μg/mL apramycin	Apramycin only	Apramycin +0.5 μg/mL tigecycline
IC_50_ (μg/mL)	0.14 ± 0.11	<0.0001	0.44 ± 0.02	<0.0001
Hill Slope	0.54 ± 0.2	1.00 ± 0.72	2.85 ± 0.35	0.70 ± 0.34
R^2^	0.7752	0.6646	0.9513	0.7722

### 3.6 *In vivo* synergistic efficacy

We next examined whether this synergism would also be apparent *in vivo* using a neutropenic mouse thigh model. The bacterial density in the control groups were maintained at 10^6^–10^7^ CFU/g after 24 h treatment with saline. In contrast, bacterial growth was reduced to 10^2^–10^3^ CFU/g using combination therapy against all four test strains (*p* < 0.001). Significant differences (*p* < 0.05) between groups of tigecycline alone and combined with apramycin were shown in Figure 1B against tested strains except WF106-1. Comparing with apramycin alone, the combined regimen showed a slight decrease in bacterial burden after 24 h treatment, but a significant bacterial reduction was only observed against strain HNS1-2 (*p* < 0.05). These data indicated that combination therapy of tigecycline and apramycin generated a synergistic antibacterial efficacy *in vivo*.

### 3.7 High-level tigecycline-resistance development

**Figure 6:**
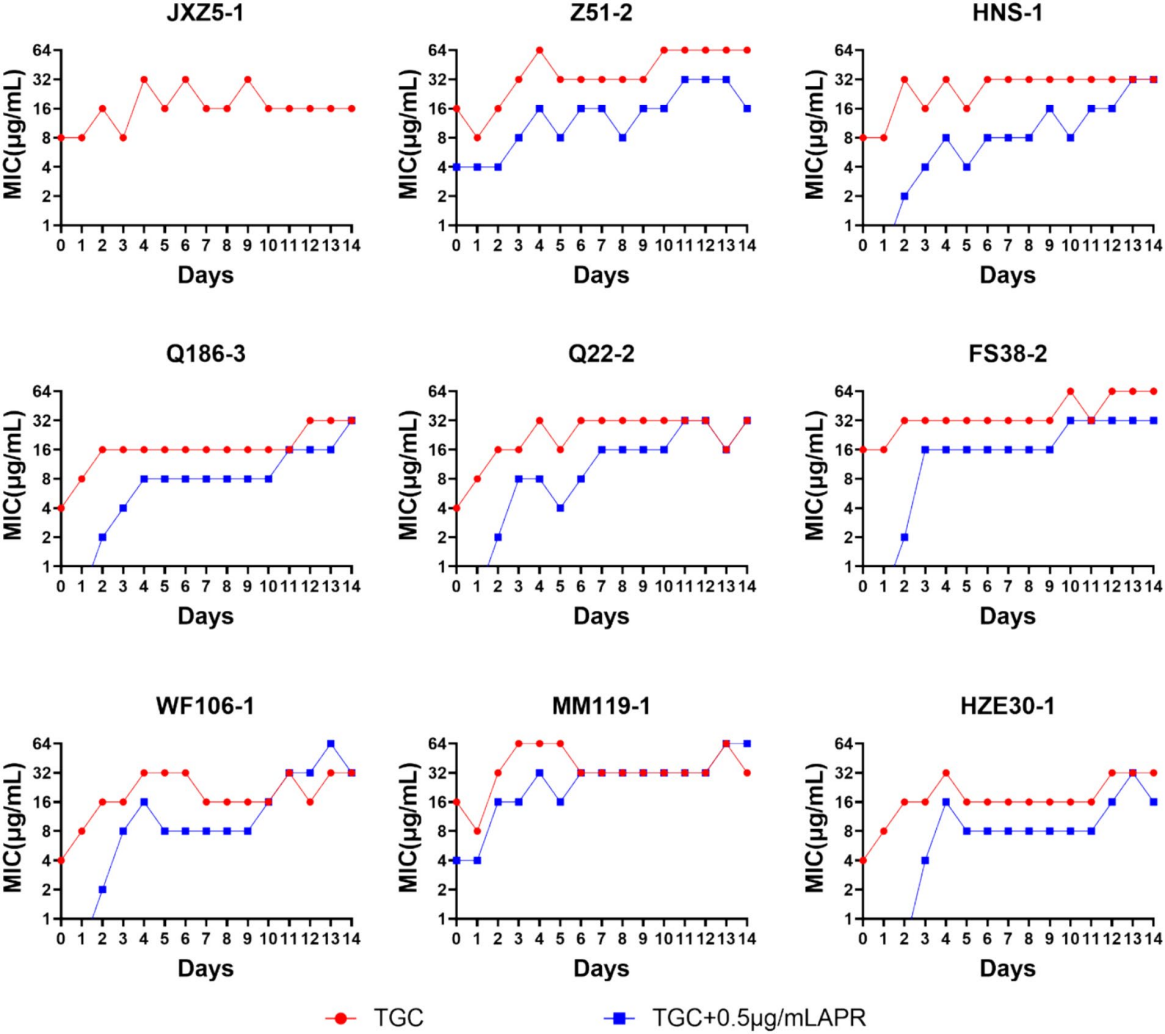
MIC_TGC_ changes of *tet*(X)-harboring *Acinetobacter* strains under drug selective pressure for 14 days. Red line, tigecycline alone; blue line, tigecycline plus 0.5 μg/mL apramycin.

When tested strains were exposed to tigecycline alone, tigecycline MICs for most of the strains showed rapid fluctuating rising trend. This was especially apparent for strains MM119-1 and Z51-2 with tigecycline MICs reached 64 μg/mL on the third or fourth day. The addition of 0.5 μg/mL apramycin slowed down the booming of tigecycline MIC, however, it would reach to 16 to 32 μg/mL after 10 days exposure. The findings indicated that strains were losing sensitivity with continuous exposure to even the combined regimen. Notably, during the 14 days of passaging only strain JXZ5-1 was completely inhibited by the combined drugs (Figure 6).

## 4 Discussion

The majority of the *tet*(X)-harboring *Acinetobacter* strains are associated with livestock, raising concerns that the tigecycline-resistance *tet*(X) genes could be transmitted to humans through opportunistic pathogens like *Acinetobacter* (Cheng et al., 2021; Chen et al., 2020; Nulsopapon et al., 2021). Previously reported, infections caused by non-fermentative Gram-negative bacilli have been treated successfully with tigecycline plus aminoglycosides (Zhou et al., 2019). Our study proved that the combination of tigecycline and apramycin was effective against *Acinetobacter* strains possessing *tet*(X) as well (Figure 2B; Supplementary Table S1).

Combination therapy has become a commonly employed approach for the treatment of severe infections caused by *Acinetobacter* spp. (Nasr, 2020). In our study, when partnered with apramycin, certain strains reverted to the tigecycline sensitive phenotype especially with the addition of 2 μg/mL apramycin and resulted in a 20-times lower in tigecycline MIC (Figure 3). The FICI and the *in vitro* time-killing curves (Figure 4) indicated that apramycin is a candidate potentiating tigecycline against *tet*(X)-harboring *Acinetobacter* strains. Dose response models in preclinical trials can be predictors of drug interactions (Kong and Lee, 2006), we found the accentuated bacteriostatic effect of tigecycline in the presence apramycin (Figure 5; Table 2). Moreover, a recent report demonstrated the potential of combining colistin/amikacin and tigecycline to achieve synergistic effects against *Acinetobacter* strains further support our findings (Wu et al., 2021).

Although the initial response of patients to a combination therapy often appears promising, the emergence of resistance to continuous therapy is common (He et al., 2020). In our group of *tet*(X)-carrying isolates, one was significantly inhibited or killed by tigecycline plus 0.5 μg/mL apramycin during the 14 days of passaging and only 22.2% of isolates possessed a high tigecycline MIC (64 μg/mL) with continuous exposure of the combination (Figure 6). This indicated that apramycin may delay the MIC increase of tigecycline.

Further confirmation of the antimicrobial activity of the combination therapy was carried out using an *in vivo* mouse model. Four strains carrying different *tet*(X) variants were colonized in the mouse thigh as expected. During the treatment, differences of 3–5 log CFU/g were achieved between control groups and the combined treatment groups and differences of 1–5 log CFU/g were observed between monotherapies and the combination therapies. Drug combination demonstrated significant antibacterial efficacy when compared to the control group (Figure 1). Previous reports have indicated that elevated MICs of either apramycin or tigecycline would dampen their clinical efficacy when utilized as a monotherapy option (Abdul-Mutakabbir et al., 2021; Sader et al., 2005; Lee et al., 2016). We found that the combination displayed a highly significant synergistic response *in vivo* against strain HNS1-2 (MIC_TGC_ = 8 μg/mL, MIC_APR_ = 4 μg/mL) carrying *tet*(X3), while tigecycline monotherapy failed to produce antibacterial outcomes. These preliminary results suggest that combinations of tigecycline with apramycin may exhibit activity against tigecycline-resistant strains.

Notably, apramycin has also been demonstrated to be of lower toxicity than other aminoglycosides (Ishikawa et al., 2019). Apramycin has completed a Phase 1 clinical trial assessing the safety, tolerability, and pharmacokinetics in healthy adults (ClinicalTrials.gov ID: NCT04105205). Given these promising findings, a combination of apramycin and tigecycline may offer a potential therapeutic option for the treatment of *Acinetobacter* infections in the future. While the present study provides promising results from both *in vitro* and *in vivo* experiments, the translation of these findings to clinical practice requires further validation. Future studies should focus on clinical trials and the use of clinical samples to assess the feasibility of applying our findings to patient populations.

Tigecycline, the first member of the glycylcycline class of antibiotics, has shown promising *in vitro* activity against multidrug-resistant strains. It binds with high affinity to bacterial ribosomes and is unaffected by the typical mechanisms that render bacteria resistant to the tetracycline class. Aminoglycosides inhibit protein synthesis by binding to the 16S rRNA and disrupt the integrity of bacterial cell membranes that may facilitate tigecycline passive accumulation (Vidaillac et al., 2012; Shakil et al., 2008). During combination therapy, tigecycline might lead to synergistic bactericidal effects by enhancing the aminoglycoside effect and inhibiting bacterial adaptive responses (Ma et al., 2022). Further research is needed to determine the exact mechanisms for the success of this combination therapy.

This study provides strong evidence that the combination of tigecycline and apramycin exhibited synergistic activity against *tet*(X)-harboring *Acinetobacter* strains. Both *in vitro* and *in vivo* results show that this combination reduces the MIC of tigecycline and enhances its bactericidal activity. Apramycin appears to delay the emergence of resistance and potentiates the efficacy of tigecycline, offering a promising strategy for treating multidrug-resistant *Acinetobacter* infections. The findings highlight the potential of this combination therapy to address the growing challenge of multidrug-resistant *Acinetobacter* spp., offering a valuable treatment option where other antibiotics may have failed.

## Data Availability

The data presented in the study are deposited in the NCBI SRA repository , under the BioProject accession number PRJNA1194363.
