# Supplementary material for: Overcoming tet(X)-harboring tigecycline resistance: a study on the efficacy of tigecycline-apramycin combinations
Source: Front Microbiol. 2024 Dec 23;15:1502558. doi: 10.3389/fmicb.2024.1502558 (PMC11701230; doi:10.3389/fmicb.2024.1502558)
Supplement: Supplementary file 1 [file Table_1.docx]

Supplementary Material

**Table S1**. Tigecycline or apramycin MICs and combined checkerboard FICI against *tet*(X)-harboring *Acinetobacter* strains.

| Strain | Resistance gene | MIC（μg/mL） | | |
| --- | --- | --- | --- | --- |
|  |  | TGC | APR | FICI |
| JXZ5-1 | *tet*(X3) | 8 | 1 | 0.313 |
| Z51-2 | *tet*(X3) | 16 | 2 | 0.750 |
| HNS1-2 | *tet*(X3) | 8 | 4 | 0.313 |
| HZE30-1 | *tet*(X3) *tet*(X6) *bla*_NDM-1_ | 4 | 2 | 0.250 |
| MM119-1 | *tet*(X3) *tet*(X6) *bla*_NDM-3_ | 16 | 4 | 0.313 |
| FS38-2 | *tet*(X3) *tet*(X6) *bla*_NDM-1_ | 16 | 2 | 0.088 |
| WF106-1 | *tet*(X3) *tet*(X6) *bla*_NDM-1_ | 4 | 4 | 0.313 |
| Q22-2 | *tet*(X4) | 4 | 2 | 0.375 |
| Q186-3 | *tet*(X4)  *tet*(X5) | 4 | 2 | 0.188 |
